# Role of endothelial dysfunction in sleep-disordered breathing in egyptian children with sickle cell disease

**DOI:** 10.1186/s12887-024-05066-6

**Published:** 2024-10-01

**Authors:** Ilham Youssry, Abla S. Mostafa, Dina H. Hamed, Yasmin F. Abdel Hafez, Irene E. Bishai, Yasmeen M. M. Selim

**Affiliations:** 1 Department of Pediatric Hematology, Faculty ofMedicine, Cairo University, Giza, Egypt; 2 Department of Pediatric Pulmonology, Faculty of Medicine, Cairo University, Giza, Egypt; 3 Department of Pediatrics, Faculty of Medicine, Cairo University, Giza, Egypt; 4 Department of Clinical and Chemical Pathology, Faculty of Medicine, Cairo University, Giza, Egypt

**Keywords:** Sickle cell disease, Sleep disordered breathing, Endothelial dysfunction

## Abstract

**Background:**

Endothelial dysfunction is an integral pathophysiologic mechanism in sickle cell disease (SCD), and can lead to many complications. Sleep-disordered breathing (SDB) is a SCD complication with diverse incidence and pathophysiology. This study aimed to determine the prevalence of SDB in children with SCD and to assess its relation to endothelial dysfunction.

**Methods:**

Sixty children with SCD and 60 healthy controls were enrolled. The levels of TNF-α, IL-6, and IL-17A were evaluated in the entire cohort using enzyme-linked immunosorbent assay (ELISA) kits. Polysomnography (PSG) was performed for all SCD patients after completion of the Pediatric Sleep Questionnaire (PSQ).

**Results:**

TNF-α, IL-6, and IL-17A levels were significantly greater in children with SCD than in controls (*p*-values < 0.001, < 0.001, and 0.006, respectively). The PSQ revealed symptoms suggestive of SDB in 50 children with SCD (83.3%), and PSG revealed obstructive sleep apnea (OSA) in 44 children with SCD (73.3%); 22 patients had mild OSA, and 22 had moderate-to-severe OSA according to the apnea–hypopnea index (AHI). TNF-α was significantly greater in SCD children who reported heavy or loud breathing, trouble breathing or struggle to breathe, and difficulty waking up in the morning (*p*-values = 0.002, 0.002, and 0.031, respectively). The IL-6 levels were significantly greater in SCD children who stopped growing normally (*p*-value = 0.002). The levels of IL-6 and IL-17A were significantly greater in SCD children with morning headaches (*p*-values = 0.007 and 0.004, respectively).

**Conclusion:**

Children with SCD showed a high prevalence of SDB with significantly elevated levels of markers of endothelial function, highlighting the interplay of SDB and endothelial dysfunction in SCD.

**Supplementary Information:**

The online version contains supplementary material available at 10.1186/s12887-024-05066-6.

## Background

Endothelial dysfunction (ED) is an important feature of sickle cell disease (SCD) [1] and is associated with several contributing factors, including wall shear stress, stiff sickle erythrocyte adhesion, and interaction with the vascular endothelium, hypoxia, hyperviscosity, and reduced nitric oxide bioavailability [2–5]. Endothelial function (EF) in SCD has been previously assessed by several methods, including the measurement of circulating extracellular vesicles (EVs), proinflammatory cytokines, endothelial-derived microparticles (EDMPs), and adhesion molecules [6–9]. Flow-mediated dilation of the brachial artery and peripheral arterial tonometry [10–12] are also among the noninvasive methods used to assess EF in SCD patients.

Various circulating biomarkers have been used previously to monitor endothelial dysfunction in human studies including inflammatory biomarkers such as high-sensitivity C-reactive protein (hs-CRP), interleukin 6 (IL-6), interleukin 8 (IL-8), interleukin 1β (IL-1β), tumor necrosis factor α (TNF-α), CC-chemokine ligand 2 (CCL2), interleukin 17 (IL-17), and others [13–17]. Activated endothelial cells in SCD patients release inflammatory cytokines, including TNF-α, IL-6, and IL-17, which have been proven to contribute to the inflammatory process and ED observed in SCD patients and hence play a pivotal role in SCD complications [18–21].

One of the frequently underdiagnosed SCD complications is sleep-disordered breathing (SDB), which encompasses obstructive sleep apnea (OSA) and nighttime hypoxemia [22]. The estimated prevalence of OSA is 41% in children with the Hb SS genotype and 10 –15% in those with less severe genotypes [23, 24]. Adenotonsillar hypertrophy (ATH) secondary to functional asplenia, recurrent tonsillitis from defective bacterial opsonization, or extramedullary hematopoiesis has been implicated in the greater incidence of OSA in children with SCD [25, 26]. Hypoxia, which enhances oxidative stress and proinflammatory signaling pathways, is a principal trigger of the pathophysiological mechanism shared by SDB and SCD and contributes to comorbidities attributed to both diseases such as cardiovascular, pulmonary, and neurologic sequelae [27].

There has been a possible interplay between endothelial dysfunction and SDB studied previously [28–30]; however, this relationship has not been previously investigated in SCD patients. Hence, in this study, we aimed to determine the prevalence of SDB in Egyptian children with SCD and assess endothelial function in such a cohort. Then, we determined the relationship between endothelial dysfunction and SDB in these children.

## Methods

### Study population

This cross-sectional study included 60 sickle cell disease (SCD) children aged 8–18 years with a mean age of 11.4 ± 2.71 years. The entire cohort enrolled was in a steady state and one month away from previous blood transfusions. The recruited patients were followed at the Pediatric Hematology Outpatient Clinic of Cairo University Children’s Hospital and diagnosed via hemoglobin electrophoresis. Forty-four (73.3%) patients were homozygous (HbSS), and 16 (26.7%) were compound heterozygous for sickle β-thalassemia (HbSβ) [3 patients were Sβ^0^, and 13 patients were Sβ^+^]. Patients with chronic disease, skeletal deformity, craniofacial anomalies, neuromuscular disorders, or acute upper respiratory tract infection 2 weeks before enrollment were excluded from this study.

Thirty-five (58.3%) of the studied SCD children were males, and 25 (41.7%) were females. The mean age at diagnosis was 21 ± 16.4 months. Regarding anthropometric measurements of the studied patients, the mean weight was 30.40 ± 11.03 kg, the mean height was 130.1 ± 18.64 cm, and the mean BMI was 17.30 ± 1.66 kg/m^2^. All our patients were receiving hydroxycarbamide at a mean daily dose of 18.9 ± 6.6 mg/kg/day, and 21.6% required iron chelation due to hyperferritinemia, with a mean serum ferritin level of 728.05 ± 995.99 ng/ml. The mean hemoglobin of the studied patients was 9.06 ± 1.21 gm/dL, the mean platelet count was 285.77 ± 121.94 × 10^3^/cmm, the mean total leukocyte count was 10.10 ± 3.9 × 10^3^/cmm, the mean HbS level was 64.36 ± 12.97%, and the mean HbF level was 13.05 ± 9.48%.

As a control for the studied cytokines, 60 healthy, age- and sex-matched children were enrolled. This study was approved by the Research Ethics Committee at the Faculty of Medicine—Cairo University (ethical clearance number 589–2021). Before patient enrollment, written informed consent and assent were obtained from the patients and their guardians.

Sleep quality was assessed by the Pediatric Sleep Questionnaire Sleep-Disordered Breathing (PSQ-SDB) Subscale and polysomnography (PSG) in the enrolled patients. The levels of the studied markers of endothelial function were measured in patients and controls. All studied variables were tested within two days of each other.

## Assessment of sleep-disordered breathing in patients with sickle cell disease

### Pediatric sleep questionnaire sleep-disordered breathing (PSQ-SDB) subscale

Patients were interviewed by the same researcher to answer a questionnaire-based survey, the PSQ-SDB subscale. The PSQ consisted of 22 parent-reported items (included in the Additional file 1). The purpose of these questions was to evaluate the symptoms of snoring, witnessed sleep apnea, difficulties in breathing during sleep, daytime sleepiness, inattention, and hyperactivity. The PSQ-SDB score ranged from zero to one. Scores ≥ 0·33 were deemed positive and highly indicative of pediatric sleep-disordered breathing [31, 32].

### Polysomnography

Overnight polysomnography (PSG) was performed for all patients at the Sleep Laboratory of Cairo University Children’s Hospital. The following parameters were recorded using a computerized recording system (Embla A10®, Embla, Broomfield, CO, USA): [33]

1. Brainwave activity: The electroencephalogram (EEG) electrodes were applied at C4, C3, O1, and O2, with A1 and A2 as reference electrodes.
2. Muscle activity: Submental and anterior tibialis electromyograms (EMGs) were used. Periodic limb movements in sleep (PLMS) were assessed using anterior tibialis EMG.
3. Eye movements: A two-channel electrooculogram (EOG) was used.
4. Heart rate and rhythm: A two-lead electrocardiogram (ECG) was used.
5. Airflow: An Embla nasal pressure cannula and a Nihon Kohden, Tokyo, Japan three-pronged thermistor were used.
6. Thoracoabdominal movements: Two respiratory inductance plethysmography belts (RIPs) were used.
7. Snoring activity: A snore microphone was used.
8. Arterial oxygen saturation (SpO2): SpO2 was determined by pulse oximetry (Masimo, Irvine, CA).
9. Body position: A body position sensor was used.

All PSG records were videotaped.

The following variables were recorded for each PSG: total sleep time (TST); sleep efficiency; sleep stages [nonrapid-eye movement (NREM) sleep (stages N1, N2, and N3) and rapid-eye movement (REM) sleep]; apnea–hypopnea index (AHI); oxygen desaturation index (ODI); and periodic limb movement index (PLMI).

The American Academy of Sleep Medicine (AASM) scoring guidelines were followed:

- **Apnea **was defined as a ≥ 90% decrease in peak signal excursion compared to the pre-event baseline, spanning a minimum of two breaths, and fulfilling respiratory effort requirements for different types of apneas. Obstructive apneas were accompanied by respiratory effort during the airflow absence period; central apneas were accompanied by the absence of respiratory effort during the airflow absence period; and mixed apneas were accompanied by the presence and absence of respiratory effort occurring during the same event, irrespective of which of them occurred first [34].
- **Hypopnea **was defined as a ≥ 30% decrease in peak signal excursion compared to the pre-event baseline, spanning a minimum of two breaths, with ≥ 3% desaturation, or linked with arousal [34].
- The apnea–hypopnea index (AHI) was calculated by recording the average number of apneas and hypopneas that occurred during an hour of sleep. Patients were categorized as having mild OSA (5 – 15 events/hour), moderate OSA (> 15 – 30 events/hour), or severe OSA (> 30 events/hour) [35].

### Assessment of endothelial function in SCD patients and control group 

Serum concentrations of TNF-α, IL-6, and IL-17A were evaluated using commercially available enzyme-linked immunosorbent assay (ELISA) kits (Sunred Biological Technology, Shanghai, China, Catalog nos. 201–12- 0083, 201–12- 0091, and 201–12- 0048, respectively) according to the manufacturer's instructions (R&D Systems, MN, USA).

### Statistical analysis

Categorical data were represented as numbers and percentages. Chi-square test was applied to compare between two groups. Alternatively, Fisher Exact correction and Monte Carlo correction tests were applied when more than 20% of the cells had an expected count of less than 5. For continuous data, they were tested for normality by the Shapiro–Wilk test. Quantitative data were expressed as range (minimum and maximum), mean, standard deviation, and median for normally distributed quantitative variables. Student t-test was used to compare two groups while one-way ANOVA test was used to compare the different studied groups. On the other hand, for not normally distributed quantitative variables, the Mann–Whitney test was used to compare two groups while the Kruskal–Wallis test was used to compare different groups. The significance of the obtained results was judged at the 5% level. A p-value ≤ 0.05 was considered statistically significant. All the statistical computations were performed using IBM SPSS (Statistical Package for the Social Science; IBM Corp., Armonk, NY, USA), release 28 for Microsoft Windows [36–38].

## Results

### Markers of endothelial function in the studied SCD patients

**Fig. 1:**
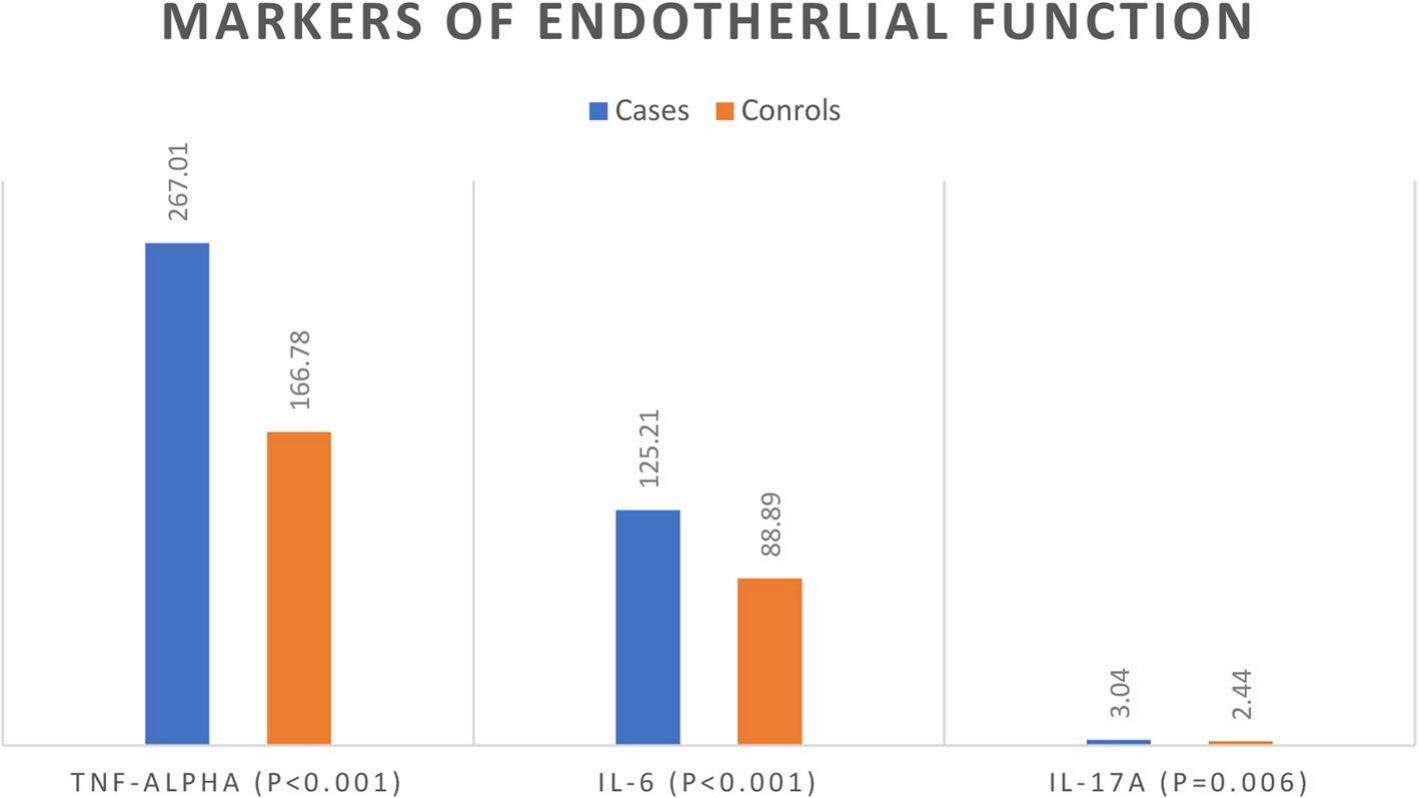
Comparison between cases and controls regarding the mean of markers of endothelial function

**Fig. 2:**
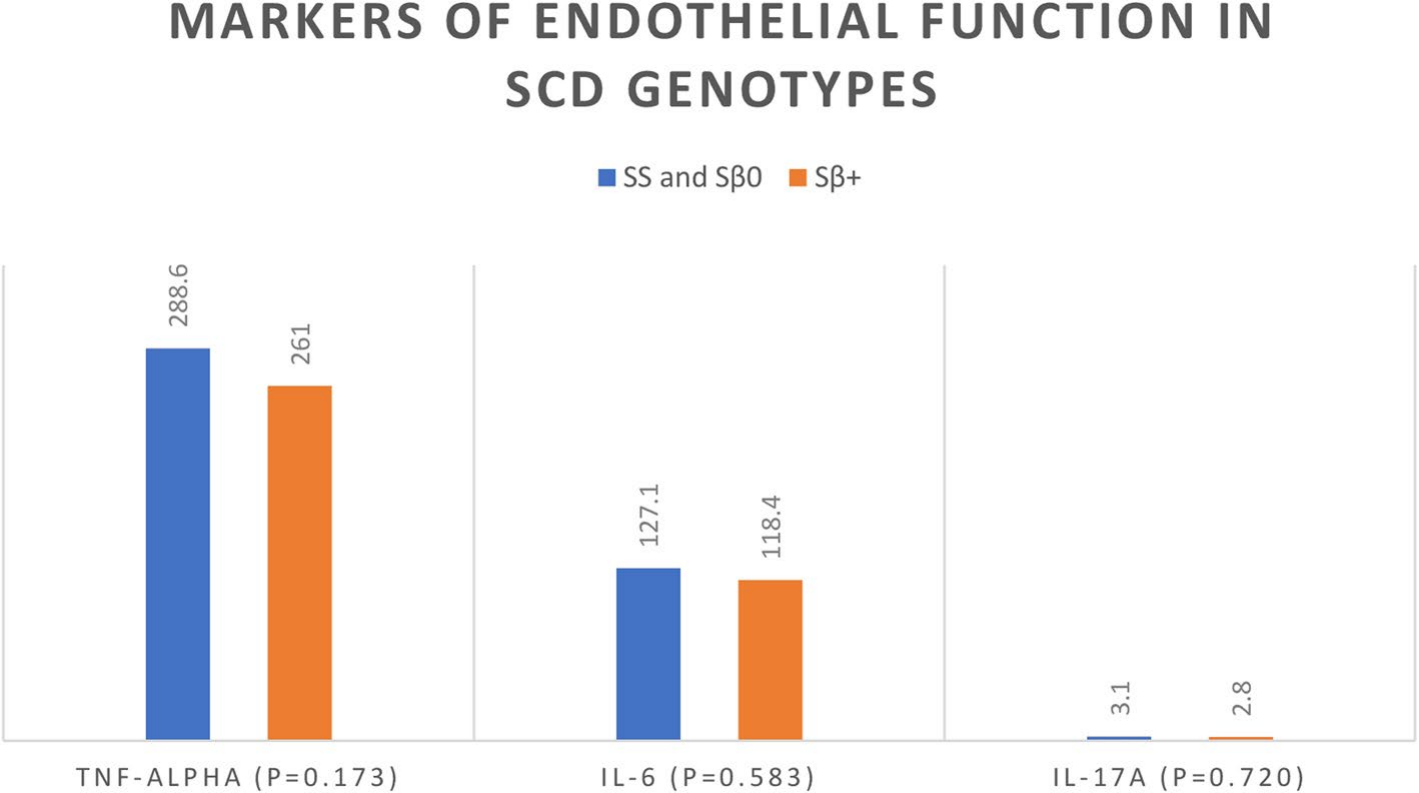
Comparison between SCD genotypes regarding the mean of markers of endothelial function

In the studied SCD patients, the mean TNF-α level was 267.01 ± 100.04 ng/L, the mean IL-6 level was 125.21 ± 50.06 ng/L, and the mean IL-17A level was 3.04 ± 2.02 ng/L. Sickle cell disease patients showed significantly higher markers of endothelial function (TNF-α, IL-6, and IL-17A) in comparison to controls (*p*-values < 0.001, < 0.001, and 0.006, respectively) (Fig. 1). Sickle cell disease patients with more severe genotypes (SS and Sβ^0^) had slightly greater levels of markers of endothelial function (TNF-α, IL-6, and IL-17A) than Sβ^+^ patients, but the difference was not statistically significant (*p*-values = 0.173, 0.583, and 0.720, respectively) (Fig. 2).

### Sleep-disordered breathing (SDB) in the studied SCD patients

According to the PSQ score (Table 1), 50 of the studied SCD patients (83.3%) displayed symptoms of sleep-disordered breathing (SDB). Regarding the symptoms assessed by the questionnaire, the most prominent symptoms were difficulty waking up in the morning, snoring, and waking up feeling unrefreshed in the morning (83.3%, 76.7%, and 76.7%, respectively).

**Table 1:** Pediatric sleep questionnaire items for assessment of pediatric sleep-related breathing disorder in SCD patients

Pediatric sleep questionnaire symptoms		The number of SCD patients showing the symptom (Total = 60)	Percentage of SCD patients showing the symptom (Total = 100%)
**Snoring more than half the time**		22	36.7%
**Always snore**		14	23.3%
**Snore loudly**		10	16.7%
**Heavy or loud breathing**		11	18.3%
**Trouble breathing or struggle to breathe**		11	18.3%
**Stop breathing during the night**		5	8.3%
**Mouth breather**		15	25%
**Dry mouth on waking up in the morning**		14	23.3%
**Wet the bed**		9	15%
**Wake up feeling unrefreshed in the morning**		46	76.7%
**Day sleepiness**		41	68.3%
**Teacher comment of a sleepy child**		39	65%
**Hard to wake up in the morning**		50	83.3%
**Morning headaches**		12	20%
**Stop growing at a normal rate at any time since birth**		21	35%
**Overweight**		0	0%
**Unattentive when spoken to**		36	60%
**Difficulty in organizing tasks and activities**		39	65%
**Easily distracted by extraneous stimuli**		41	68.3%
**Fidgets with hands or feet or squirms in Seat**		34	56.7%
**On the go**		37	61.7%
**Interrupts or intrudes on others**		32	53.3%
**PSQ Score**	**Normal (< 0.33)**	10	16.7%
	**Abnormal (≥ 0.33)**	50	83.3%

*PSQ* Pediatric sleep questionnaire, *SCD* Sickle cell disease

 Based on polysomnography (Table 2), 44 out of 60 SCD children (73.3%) had obstructive sleep apnea (OSA); 22 (36.7%) had mild OSA, and 22 (36.7%) had moderate-to-severe OSA. For the studied patients, the AHI ranged from 0.2 to 40 occurrences per hour of sleep, with a mean of 2.47 ± 1.39 for the no OSA group, 10.2 ± 3.21 for the mild OSA group, and 21.15 ± 9.33 for the moderate-to-severe OSA group.

**Table 2:** Comparison between patients with and without OSA; and patients with mild and moderate-to-severe OSA regarding PSG parameters

**PSG parameters [Mean ± SD]**	**Non-OSA** **(*n*= 16)**	**OSA** **(*n* = 44)**		***p***
TST (min)	355.34 ± 63.49	314.60 ± 85.72		0.089
Sleep efficiency (%)	86.89 ± 10.35	77.31 ± 18.20		0.014*
NREM 1 (%)	21.81 ± 17.91	17.99 ± 10.46		0.987
NREM 2 (%)	35.22 ± 23.89	28.36 ± 22.10		0.300
NREM 3 (%)	36.13 ± 9.33	43.74 ± 19.88		0.242
REM (%)	6.72 ± 7.60	9.86 ± 15.03		0.476
ODI	0.73 ± 0.78	2.93 ± 4.93		0.064
PLMI	3.94 ± 3.81	7.46 ± 9.64		0.325
AHI	2.47 ± 1.39	15.67 ± 8.84		< 0.001*
Lowest nocturnal O_2_ saturation %	85.25 ± 7.90	72.82 ± 6.91		< 0.001*
PSG parameters [Mean ± SD]		**Mild** **OSA** **(*n*= 22)**	**Moderate-Severe** **OSA** **(*n* = 22)**	***p***
TST (min)		349.93 ± 77.55	279.27 ± 80.11	0.005*
Sleep efficiency (%)		86.71 ± 12.82	67.90 ± 18.12	< 0.001*
NREM 1 (%)		20.65 ± 12.12	15.32 ± 7.91	0.092
NREM 2 (%)		33.37 ± 22.18	23.35 ± 21.35	0.134
NREM 3 (%)		37.28 ± 16.45	50.19 ± 21.25	0.030*
REM (%)		8.62 ± 6.21	11.10 ± 20.51	0.591
ODI		3.36 ± 6.33	2.50 ± 3.04	0.565
PLMI		6.47 ± 9.03	8.45 ± 10.32	0.501
AHI		10.20 ± 3.21	21.15 ± 9.33	< 0.001*
Lowest nocturnal O_2_ saturation %		72.64 ± 7.21	73.00 ± 6.67	0.864

*OSA* Obstructive sleep apnea, *SCD* Sickle cell disease, *PSG* Polysomnography, *SD* Standard deviation, *TST* Total sleep time, *NREM* non-rapid eye movement, *REM* Rapid eye movement, *ODI* Oxygen desaturation index, *PLMI* Periodic limb movement index, *AHI* Apnea–hypopnea index

*p p*-value for comparing between the two studied groups

^*^Statistically significant at *p* ≤ 0.05

Polysomnography revealed significantly lower sleep efficiency and nocturnal oxygen saturation in SCD patients with OSA than in those without OSA (*p*-values = 0.014 and < 0.001, respectively). Regarding the severity of OSA, total sleep time and sleep efficiency were significantly lower in the moderate-to-severe OSA patients than in the mild OSA patients (*p*-values = 0.005 and < 0.001, respectively) (Table 2).

### Markers of endothelial function and SDB in the studied SCD patients

TNF-α was significantly greater in SCD patients who experienced heavy or loud breathing, trouble breathing, or struggle to breathe and who had difficulty waking up in the morning (*p*-values = 0.002, 0.002, and 0.031, respectively). IL-6 was significantly greater in SCD patients who stopped growing at a normal rate and those with morning headaches (*p*-values = 0.002 and 0.007, respectively). IL-17A was significantly greater in SCD patients with morning headaches (*p*-value = 0.004) (Table 3).

**Table 3:** PSQ items with significant positive variations in markers of endothelial function in SCD patients

	**Heavy or loud breathing**						***p***
	**Yes (= 11)**			**No (= 49)**			
	**Mean ± SD**	**Min**	**Max**	**Mean** ± **SD**	**Min**	**Max**	
**TNF-α (ng/L)**	341.07 ± 81.69	221.50	500.14	250.39 ± 96.81	11.35	644.23	0.002*
**IL-6 (ng/L)**	149.39 ± 60.89	81.06	260.12	119.78 ± 46.30	29.50	254.80	0.095
**IL-17A (ng/L)**	3.73 ± 2.42	0.98	9.53	2.89 ± 1.91	0.72	9.52	0.225
	**Trouble breathing or struggle to breathe**						
	**Yes (= 11)**			**No (= 49)**			
	**Mean ± SD**	**Min**	**Max**	**Mean** ± **SD**	**Min**	**Max**	
**TNF-α (ng/L)**	341.07 ± 81.69	221.50	500.14	250.39 ± 96.81	11.35	644.23	0.002*
**IL-6 (ng/L)**	149.39 ± 60.89	81.06	260.12	119.78 ± 46.30	29.50	254.80	0.095
**IL-17A (ng/L)**	3.73 ± 2.42	0.98	9.53	2.89 ± 1.91	0.72	9.52	0.225
	**Hard to wake up in the morning**						
	**Yes (= 50)**			**No (= 10)**			
	**Mean ± SD**	**Min**	**Max**	**Mean** ± **SD**	**Min**	**Max**	
**TNF-α (ng/L)**	281.68 ± 94.41	139.03	644.23	193.67 ± 99.53	11.35	301.78	0.031*
**IL-6 (ng/L)**	128.43 ± 52.56	29.50	260.12	109.07 ± 32.34	37.07	153.65	0.736
**IL-17A (ng/L)**	3.16 ± 2.13	0.76	9.53	2.45 ± 1.19	0.72	4.07	0.539
	**Morning Headache**						
	**Yes (= 12)**			**No (= 48)**			
	**Mean** ± **SD**	**Min**	**Max**	**Mean** ± **SD**	**Min**	**Max**	
**TNF-α (ng/L)**	278.22 ± 139.84	11.35	500.14	264.21 ± 89.15	77.05	644.23	0.691
**IL-6 (ng/L)**	163.65 ± 59.31	78.87	254.80	115.60 ± 43.03	29.50	260.12	0.007*
**IL-17A (ng/L)**	4.52 ± 2.38	2.14	9.52	2.68 ± 1.76	0.72	9.53	0.004*
	**Stop Growing at a normal rate at any time since birth**						
	**Yes (= 21)**			**No (= 39)**			
	**Mean ± SD**	**Min**	**Max**	**Mean** ± **SD**	**Min**	**Max**	
**TNF-α (ng/L)**	267.88 ± 112.37	11.35	500.14	266.54 ± 94.31	77.05	644.23	0.739
**IL-6 (ng/L)**	146.14 ± 49.71	78.87	254.80	113.94 ± 47.10	29.50	260.12	0.002*
**IL-17A (ng/L)**	3.59 ± 2.18	0.72	9.52	2.75 ± 1.88	0.76	9.53	0.088

*PSQ* Pediatric sleep questionnaire, *SCD* Sickle cell disease, *TNF-α* Tumor necrosis factor-alpha, *IL* Interleukin, *SD* Standard deviation, *Min.* Minimum, *Max*. Maximum

*p p*-value for comparing between the two studied groups

^*^Statistically significant at *p* ≤ 0.05

The levels of markers of endothelial function (TNF-α and IL-6) were slightly greater but not significantly different between patients with and without OSA (*p*-values = 0.953 and 0.817, respectively). Obstructive sleep apnea was more prevalent among males (*p*-value = 0.01). Sickle cell disease patients with OSA had a greater HbS level and a greater total leukocyte count (*p*-values = 0.019 and 0.047, respectively) (Table 4).

**Table 4:** Comparison between SCD patients with and without OSA regarding demographic, clinical, and laboratory parameters, and PSQ score

			**Non-OSA** **(*n* = 16)**	**OSA** **(*n* = 44)**	***p***
**Gender**		**Male**	5 (31.3%)	30 (68.2%)	0.01*
		**Female**	11 (68.7%)	14 (31.8%)	
**Age at enrollment (years) [Mean ± SD]**			10.44 ± 2.60	111.75 ± 2.69	0.097
**Blood transfusion frequency in the preceding 12 months [Mean ± SD]**			2.25 ± 3.13	3.09 ± 4.18	0. 469
**SCD genotypes*n*(%)**					
** SS**			13 (81.3%)	31 (70.4%)	0.294
** Sβ**^0^			2 (12.5%)	1 (2.2%)	
** Sβ**^+^			1 (6.3%)	12 (27.2%)	
**Hydroxyurea dose in mg/kg/day [Mean ± SD]**			20.8 ± 8.2	18.2 ± 5.9	0.753
**Laboratory Parameters**					
** HbS (%)**	**Mean ± SD**		58.1 ± 10.6	66.6 ± 13.1	0.019^*^
	**Median (Min. – Max.)**		57.4 (40 – 77)	67.6 (31.7 – 98)	
** HbA1 (%)**	**Mean ± SD**		23.4 ± 13.2	19 ± 14.8	0.152
	**Median (Min. – Max.)**		25.2 (1.80 – 47)	16.6 (0 – 55.4)	
** HbF (%)**	**Mean ± SD**		15 ± 10.8	12.4 ± 9	0.569
	**Median (Min. – Max.)**		11.6 (1.30 – 39.7)	11.1 (0 – 33.6)	
** TLC (× 10**^3^/cmm)	**Mean ± SD**		8.4 ± 3.4	10.7 ± 3.9	0.047^*^
	**Median (Min. – Max.)**		7.5 (4.39 – 13.6)	10.6 (4.60 – 24)	
** N/L Ratio**	**Mean ± SD**		0.8 ± 0.1	0.9 ± 0.4	0.273
	**Median (Min. – Max.)**		0.8 (0.58 – 1.2)	0.8 (0 – 2.2)	
** Hb (gm/dL)**	**Mean ± SD**		9.4 ± 1.3	8.9 ± 1.2	0.057
	**Median (Min. – Max.)**		9.6 (5.80 – 11.1)	9 (6.80 – 12.9)	
** PLT (× 10**^3^/cmm)	**Mean ± SD**		253.1 ± 78.7	297.6 ± 133	0.542
	**Median (Min. – Max.)**		277 (123 – 365)	265.5 (117 – 693)	
** LDH (u/L)**	**Mean ± SD**		441.9 ± 159.9	451.9 ± 85	0.622
	**Median (Min. – Max.)**		427.5 (178 – 900)	450 (324 – 640)	
** CRP (mg/L)**	**Mean ± SD**		4.7 ± 1.5	5.6 ± 1.5	0.075
	**Median (Min. – Max.)**		5 (2 – 7)	6 (2 – 9)	
** Reticulocyte count (%)**	**Mean ± SD**		1.2 ± 0.7	1.3 ± 0.8	0.906
	**Median (Min. – Max.)**		1 (0.10 – 2)	1 (0 – 3)	
** Indirect bilirubin (mg/dL)**	**Mean ± SD**		1.3 ± 0.7	1.1 ± 0.7	0.425
	**Median (Min. – Max.)**		1.2 (0.20 – 2.5)	1 (0.30 – 3)	
** Serum Ferritin (ng/mL)**	**Mean ± SD**		601.5 ± 866.1	774.1 ± 1044.7	0.980
	**Median (Min. – Max.)**		267.4 (113.8 – 3643)	271.2 (105 – 3987)	
**Markers of endothelial function**					
** TNF-α (ng/L)**	**Mean ± SD**		263.9 ± 71.4	275.7 ± 157	0.953
	**Median (Min. – Max.)**		262.1 (137 – 440.5)	267.3 (11.35 – 644.2)	
** IL-6 (ng/L)**	**Mean ± SD**		124.3 ± 45.6	127.7 ± 62.4	0.817
	**Median (Min. – Max.)**		115.3 (29.5 – 260.1)	117.6 (377 – 254.8)	
** IL-17A (ng/L)**	**Mean ± SD**		3.1 ± 1.9	3 ± 2.3	0.581
	**Median (Min. – Max.)**		2.6 (0.72 – 9.5)	2.7 (0.76 – 9.5)	
** PSQ score **	**Mean ± SD**		0.38 ± 0.16	0.40 ± 0.11	0.900
	**Median (Min. – Max.)**		0.4 (0.09 - 0.72)	0.38 (0.22 – 0.6)	

*OSA* Obstructive sleep apnea, *SCD* Sickle cell disease, *SD* Standard deviation, *VOC* Vaso-occlusive crisis, *HbS* Sickle hemoglobin, *HbA1* Major adult hemoglobin, *HbF* Fetal hemoglobin, *TLC* Total leukocyte count, *N/L Ratio *Neutrophil-to-Lymphocyte ratio, *Hb* Hemoglobin, *PLT* Platelet count, *LDH* Lactate dehydrogenase, *CRP* C-reactive protein, *TNF-α* Tumor necrosis factor-alpha, *IL* Interleukin, *PSQ* Pediatric sleep questionnaire

p:* p*-value for comparing between the two studied group

^*^Statistically significant at *p* ≤ 0.05

On comparing patients with mild OSA and moderate-to-severe OSA, we found that TNF-α, IL-6, and IL-17A were also slightly greater in the moderate-to-severe OSA patients than in the mild OSA patients, yet the difference was not statistically significant (*p*-values = 0.716, 0.404, and 0.673, respectively). Patients with moderate-to-severe OSA had a significantly lower hemoglobin level and a significantly greater platelet count than those with mild OSA (*p*-values = 0.035 and 0.036, respectively) (Table 5).

**Table 5:** Comparison between SCD patients with mild and moderate-to-severe OSA regarding demographic, clinical, and laboratory parameters, and PSQ score

		**Mild OSA** **(*n*= 22)**	**Moderate-Severe OSA** **(***n***= 22)**	***p***
**Gender**	**Male**	18 (81.8%)	12 (54.5%)	0.052
	**Female**	4 (18.2%)	10 (45.5%)	
**Age at enrollment (years) [Mean ± SD]**		11.5 ± 2.8	12.1 ± 2.4	0.391
**Blood transfusion frequency in the preceding 12 months [Mean ± SD]**		2.6 ± 4	3.6 ± 4.4	0. 362
**SCD genotypes *n* (%)**				
** SS and Sβ**^**0**^		16 (72.7%)	19 (86.4%)	0.475
** Sβ**^**+**^		6 (27.3%)	3 (13.6%)	
**Hydroxyurea dose in mg/kg/day [Mean ± SD]**		19.1 ± 7.3	17.3 ± 4.1	0.569
**Laboratory Parameters**				
** HbS (%)**	**Mean ± SD**	65.8 ± 8.8	67.5 ± 16.6	0.445
	**Median (Min. – Max.)**	65.9 (50.9 – 79.4)	69.3 (31.7 – 98)	
** HbA1 (%)**	**Mean ± SD**	19.2 ± 13.6	18.8 ± 16.2	0.580
	**Median (Min. – Max.)**	18.2 (0 – 38.2)	13 (0 – 55.4)	
** HbF (%)**	**Mean ± SD**	12.3 ± 9.1	12.4 ± 9.2	0.934
	**Median (Min. – Max.)**	11.1 (0 – 33.6)	10 (0 – 29)	
** TLC (× 10**^**3**^**/cmm)**	**Mean ± SD**	9.4 ± 2.8	12 ± 4.5	0.069
	**Median (Min. – Max.)**	9.8 (4.60 – 13.6)	11.1 (4.8 – 24)	
** N/L Ratio**	**Mean ± SD**	1 ± 0.4	0.9 ± 0.5	0.630
	**Median (Min. – Max.)**	0.8 (0.45 – 2)	0.8 (0 – 2.2)	
** Hb (gm/dL)**	**Mean ± SD**	9.4 ± 1.1	8.5 ± 1.1	0.035*
	**Median (Min. – Max.)**	9 (8 – 12.9)	8.8 (6.8 – 10.9)	
** PLT (× 10**^**3**^**/cmm)**	**Mean ± SD**	258.9 ± 111.2	336.4 ± 144	0.036*
	**Median (Min. – Max.)**	239.5 (117 – 580)	287.5 (145 – 693)	
** LDH (u/L)**	**Mean ± SD**	442.1 ± 80.7	461.6 ± 89.9	0.431
	**Median (Min. – Max.)**	418 (325 – 640)	452.5 (324 – 638)	
** CRP (mg/L)**	**Mean ± SD**	5.7 ± 1.9	5.4 ± 1.1	0.582
	**Median (Min. – Max.)**	6 (2 – 9)	6 (3 – 7)	
** Reticulocyte count (%)**	**Mean ± SD**	1.2 ± 0.8	1.4 ± 0.9	0.560
	**Median (Min. – Max.)**	1 (0 – 3)	1 (0.2 – 3)	
** Indirect bilirubin (mg/dL)**	**Mean ± SD**	1.2 ± 0.8	1 ± 0.6	0.251
	**Median (Min. – Max.)**	1.3 (0.30 – 3)	0.9 (0.3 – 2.5)	
** Serum Ferritin (ng/mL)**	**Mean ± SD**	715.4 ± 856	832.8 ± 1222.6	0.589
	**Median (Min. – Max.)**	236.5 (110 – 2785)	287 (105 – 3987)	
**Markers of endothelial function**				
** TNF-α (ng/L)**	**Mean ± SD**	260.2 ± 60.1	267.6 ± 82.5	0.716
	**Median (Min. – Max.)**	265.1 (137 – 373.8)	257.4 (139 – 440.5)	
** IL-6 (ng/L)**	**Mean ± SD**	118.4 ± 25.4	130.1 ± 59.5	0.404
	**Median (Min. – Max.)**	115.3 (81.1 – 187)	114.8 (29.5– 260.1)	
** IL-17A (ng/L)**	**Mean ± SD**	2.6 ± 0.68	3.5 ± 2.6	0.673
	**Median (Min. – Max.)**	2.6 (0.98 – 4.1)	2.7 (0.72 – 9.5)	
** PSQ score**	**Mean ± SD**	0.34 ± 0.20	0.4 ± 0.1	0.091
	**Median (Min. – Max.)**	0.3 (0.09 – 0.72)	0.4 (0.22 – 0.7)	

*OSA* Obstructive sleep apnea, *SCD* Sickle cell disease, *SD* Standard deviation, *VOC* Vaso-occlusive crisis, *HbS* Sickle hemoglobin, *HbA1* Major adult hemoglobin, *HbF* Fetal hemoglobin, *TLC* Total leukocyte count, *N/L Ratio* Neutrophil-to-Lymphocyte ratio, *Hb* Hemoglobin, *PLT* Platelet count, *LDH* Lactate dehydrogenase, *CRP* C-reactive protein, *TNF-α* Tumor necrosis factor-alpha, *IL* Interleukin, *PSQ* Pediatric sleep questionnaire

p:* p*-value for comparing between the two studied group

^*^Statistically significant at *p* ≤ 0.05

## Discussion

Endothelial dysfunction has been implicated in the pathophysiological mechanism of sickle vasculopathy and subsequently in the development of acute and chronic SCD complications [39]. Sleep-disordered breathing (SDB), particularly obstructive sleep apnea, is a relatively common but overlooked complication of SCD. The heightened likelihood of developing SDB in young patients with SCD highlights the necessity for a deeper comprehension of the connection between the pathophysiology of SDB and SCD. One theory is that both SDB and SCD display vascular endothelial dysfunction, which can happen through different pathways comprising hypoxemia, systemic inflammation, and reactive oxygen species (ROS) production [27].

Elevated serum levels of markers of endothelial function (TNF-α, IL-6, and IL-17A) have been demonstrated to be significantly greater in SCD patients than in healthy controls. Several earlier studies have shown a similar cytokine profile in SCD patients, and these elevated proinflammatory cytokines have been associated with SCD complications, including SDB [40–45].

Sleep-disordered breathing is described as a continuum of severity, ranging from partial upper airway obstruction causing snoring to total obstruction generating obstructive sleep apnea (OSA) [46]. In this study, nearly two-thirds (73.3%) of the studied SCD patients had OSA, with approximately one-third (36.7%) having moderate-severe OSA. An even greater percentage of the studied patients (83.3%) had SDB-suggestive symptoms according to the pediatric sleep questionnaire (PSQ). The PSQ-SDB subscale exhibited good diagnostic value and was useful for screening patients for OSA [47]. It has also been used in many research studies, and many additional peer-reviewed publications have added evidence of validity and provided data to indicate usefulness in predicting some consequences of pediatric OSA, such as hyperactive behavior and sleepiness, and evaluating their response to OSA treatment [48]. In light of these findings, polysomnography (PSG) is recommended for all SCD patients displaying symptoms suggestive of SDB for early diagnosis and proper treatment of OSA, which is essential because OSA may increase the risk of developing neurological dysfunction, cognitive impairment, and cardiovascular disease [49].

In this study, there was a higher prevalence of OSA in he studied SCD patients than in previous studies, in which the prevalence was estimated to range between 10 and 41% [24, 50]. However, the estimated OSA prevalence in Egyptian SCD children is not quite known due to the limited number of studies addressing this issue. An explanation for this disparity between our findings and those of other studies may be related to the severity of the SCD genotype (73.3% of our patients had Hb SS), the greater proportion displaying SDB-suggestive symptoms increasing the risk of having sleep apnea, and our small sample size may have led to discrepancies in the proportion of patients with OSA. However, higher prevalences of OSA in SCD patients (67% and 69%) have been previously reported in some studies [51, 52].

A male preponderance was observed in our OSA group (68.2%, *p*-value = 0.01). Due to anatomical variations in the pharyngeal and upper airway anatomy, several studies have found a robust association between male sex and an elevated risk of OSA in adults [53–55]. However, among children, some studies did not support this finding [56, 57]. The discrepancy in OSA prevalence between sexes might be attributed to differences in fat distribution, upper airway length and collapsibility, neurochemical regulatory mechanisms, and the arousal response [58].

The majority of the studied SCD patients with OSA had homozygous sickle cell anemia (HbSS; 70.4%), which may explain the higher level of sickle hemoglobin (HbS) in the OSA group. A similar finding of a greater prevalence of OSA among patients with the more severe genotype (HbSS) was previously reported [59].

The total leukocyte count (TLC) is an inflammatory indicator, and inflammation is a hallmark of both SCD and OSA, which explains the higher TLC in the OSA group. It is well established that SCD and OSA patients have an elevated TLC, and in SCD, it has been correlated with disease severity and a greater risk of complication development [60–63]. The increased platelet count in the moderate-severe OSA group may be related to the mix of pathophysiological events in SCD and OSA, with increased coagulability and alterations in hemostasis culminating in a pro-thrombotic state [64]. The chronic inflammatory state in both diseases may also result in reactive thrombocytosis as a result of increased levels of IL-1β and IL-6 [65].

The pathophysiology of OSA is known to be significantly influenced by chronic systemic inflammation. Various studies have previously reported elevated levels of proinflammatory cytokines, including TNF-α, IL-6, IL-8, adhesion molecules, and C-reactive protein (CRP), in children with OSA [66]. TNF-α is a proinflammatory cytokine that regulates physiological sleep and was initially associated with disorders of excessive daytime sleepiness (EDS) [67]. However, subsequent studies demonstrated its elevation in patients with OSA independent of EDS [68]. It has been used as a valuable indicator of the occurrence and development of OSA, as well as to evaluate its severity [69, 70]. Interleukin-17 (IL-17), a proinflammatory cytokine derived from T cells, was also reported to be elevated in pediatric OSA patients, indicating T-cell activation in response to inflammation [71, 72].

In this study, there were no statistically significant differences in the levels of the studied markers of endothelial function (TNF-α, IL-6, and IL-17A) between the OSA and non-OSA groups; however, SCD patients who displayed some of the symptoms suggestive of SDB had increased levels of the studied markers. Symptoms suggestive of SDB, such as snoring, repeated night awakening, difficulty falling asleep, and day sleepiness, have been reported by SCD children in previous studies [26, 73, 74]. These findings highlight the role played by endothelial dysfunction and inflammatory pathways in the pathophysiology of SDB in SCD patients.

## Conclusion

There was a high prevalence of SDB in SCD children. Additionally, endothelial dysfunction was significantly greater in children with SCD. Sleep-disordered breathing was associated with the measured inflammatory cytokines, which may highlight the interplay between SDB and endothelial dysfunction in SCD patients.

### Strength and limitations

Sleep-disordered breathing is a common, however, an overlooked problem in SCD. That’s why, all SCD patients should be evaluated for OSA, and PSG should be performed on patients with OSA symptoms. Previous research has investigated the hypothesis of a possible interaction between endothelial dysfunction and SDB. However, prior research on this interaction in SCD patients has not been conducted.

Limitations to our work include the cross-sectional nature of the study and the use of a convenient but small sample size. It is possible that a larger prospective study would have resulted in more significant associations. It would also be ideal to replicate this research work with an additional number of SCD patients in both steady and crisis states. The limited number of assessed markers of endothelial function because of the constrained resources was another limitation. Due to the relatively short time frame over which the study took place and the constrained resources, it would have been ideal to re-assess the markers of endothelial function after OSA treatment to study the impact of treatment on these markers.

## Supplementary Information

Supplementary Material 1

## Data Availability

The datasets used and/or analyzed during the current study are available from the corresponding author upon reasonable request.

## Abbreviations

SCD: Sickle cell disease
SDB: Sleep-disordered breathing
TNF-α: Tumor necrosis factor-alpha
IL: Interleukin
ELISA: Enzyme-linked immunosorbent assay
PSG: Polysomnography
PSQ: Pediatric sleep questionnaire
OSA: Obstructive sleep apnea
AHI: Apnea Hypopnea index
ED: Endothelial dysfunction
EF: Endothelial function
EVs: Extracellular vesicles
EDMPs: Endothelial-derived microparticles
CO: Colorado
USA: United States of America
EEG: Electroencephalogram
EMG: Electromyogram
PLMS: Periodic limb movements in sleep
EOG: Electrooculogram
ECG: Electrocardiogram
RIP: Respiratory inductance plethysmography
TST: Total sleep time
NREM: Nonrapid-eye movement
PLMI: Periodic limb movement index
ODI: Oxygen desaturation index
AASM: American Academy of Sleep Medicine
MN: Minnneapolis
SD: Standard deviation
IPM: International Business Machine Corporation
SPSS: Statistical Package for the Social Science
NY: New York
ROS: Reactive oxygen species
TLC: Total leukocyte count

## References

[1] de Montalembert M, Aggoun Y, Niakate A, Szezepanski I, Bonnet D. Endothelial-dependent vasodilation is impaired in children with sickle cell disease. Haematologica. 2007;92(12):1709–10. 18055999 10.3324/haematol.11253

[2] Zawar SD, Vyawahare MA, Nerkar M, Jawahirani AR. Non-invasive detection of endothelial dysfunction in sickle cell disease by Doppler ultrasonography. J Assoc Physicians India. 2005;53:677–80. 16398074

[3] Gualandro SFM, Fonseca GHH, Gualandro DM. Complicações cardiopulmonares das doenças falciformes. Rev Bras Hematol Hemoter. 2007;29(3):291–8.

[4] Reiter CD, Gladwin MT. An emerging role for nitric oxide in sickle cell disease vascular homeostasis and therapy. Curr Opin Hematol. 2003;10(2):99–107. 12579034 10.1097/00062752-200303000-00001

[5] Akinsheye I, Klings ES. Sickle cell anemia and vascular dysfunction: The nitric oxide connection. J Cell Physiol. 2010;224(3):620–5. 20578237 10.1002/jcp.22195

[6] Lapping-Carr G, Gemel J, Mao Y, Beyer EC. Circulating Extracellular Vesicles and Endothelial Damage in Sickle Cell Disease. Front Physiol. 2020;11: 1063. 33013455 10.3389/fphys.2020.01063 PMC7495019

[7] Kato GJ, Martyr S, Blackwelder WC, Nichols JS, Coles WA, Hunter LA, et al. Levels of soluble endothelium-derived adhesion molecules in patients with sickle cell disease are associated with pulmonary hypertension, organ dysfunction, and mortality. Br J Haematol. 2005;130(6):943–53. 16156864 10.1111/j.1365-2141.2005.05701.x PMC2065864

[8] Conran N, Belcher JD. Inflammation in sickle cell disease. Clin Hemorheol Microcirc. 2018;68(2–3):263–99. 29614637 10.3233/CH-189012 PMC6314308

[9] Romana M, Connes P, Key NS. Microparticles in sickle cell disease. Clin Hemorheol Microcirc. 2018;68(2–3):319–29. 29614639 10.3233/CH-189014

[10] Rees DC, Gibson JS. Biomarkers in sickle cell disease. Br J Haematol. 2012;156(4):433–45. 22122125 10.1111/j.1365-2141.2011.08961.x

[11] Ayoola OO, Bolarinwa RA, Onwuka CC, Idowu BM, Aderibigbe AS. Association between endothelial dysfunction, biomarkers of renal function, and disease severity in sickle cell disease. Kidney360. 2020;1(2):79–85. 35372907 10.34067/KID.0000142019 PMC8809097

[12] Sivamurthy KM, Dampier C, MacDermott M, Maureen M, Cahill M, Hsu LL. Peripheral arterial tonometry in assessing endothelial dysfunction in pediatric sickle cell disease. Pediatr Hematol Oncol. 2009;26(8):589–96. 19954369 10.3109/08880010903271689

[13] Sena CM, Gonçalves L, Seiça R. Methods to evaluate vascular function: a crucial approach towards predictive, preventive, and personalised medicine. EPMA J. 2022;13(2):209–35. 35611340 10.1007/s13167-022-00280-7 PMC9120812

[14] Dri E, Lampas E, Lazaros G, Lazarou E, Theofilis P, Tsioufis C, Tousoulis D. Inflammatory Mediators of Endothelial Dysfunction. Life (Basel). 2023;13(6):1420. 37374202 10.3390/life13061420 PMC10305352

[15] Zhang J. Biomarkers of endothelial activation and dysfunction in cardiovascular diseases. Rev Cardiovasc Med. 2022;23(2):73. 35229564 10.31083/j.rcm2302073

[16] Drenjancevic I, Jukic I, Stupin A, Cosic A, Stupin M, Selthofer-Relatic K. The markers of endothelial activation. In: Helena Lenasi, editor. Endothelial dysfunction - old concepts and new challenges. 2018. 10.5772/intechopen.74671.

[17] Marder W, Khalatbari S, Myles JD, Hench R, Yalavarthi S, Lustig S, Brook R, Kaplan MJ. Interleukin 17 as a novel predictor of vascular function in rheumatoid arthritis. Ann Rheum Dis. 2011;70(9):1550–5. 21727237 10.1136/ard.2010.148031 PMC3151670

[18] Qari MH, Dier U, Mousa SA. Biomarkers of inflammation, growth factor, and coagulation activation in patients with sickle cell disease. Clin Appl Thromb Hemost. 2012;18(2):195–200. 21949038 10.1177/1076029611420992

[19] Vilas-Boas W, Cerqueira BA, Zanette AM, Reis MG, Barral-Netto M, Goncalves MS. Arginase levels and their association with Th17-related cytokines, soluble adhesion molecules (sICAM-1 and sVCAM-1) and hemolysis markers among steady-state sickle cell anemia patients. Ann Hematol. 2010;89(9):877–82. 20405289 10.1007/s00277-010-0954-9 PMC2908460

[20] Pathare A, Al Kindi S, Alnaqdy AA, Daar S, Knox-Macaulay H, Dennison D. Cytokine profile of sickle cell disease in Oman. Am J Hematol. 2004;77(4):323–8. 15551290 10.1002/ajh.20196

[21] Bandeira IC, Rocha LB, Barbosa MC, Elias DB, Querioz JA, Freitas MV, et al. Chronic inflammatory state in sickle cell anemia patients is associated with HBB(*)S haplotype. Cytokine. 2014;65(2):217–21. 24290434 10.1016/j.cyto.2013.10.009

[22] Marcus CL, Brooks LJ, Draper KA, Gozal D, Halbower AC, Jones J, et al. Diagnosis and management of childhood obstructive sleep apnea syndrome. Pediatrics. 2012;130(3):e714–55. 22926176 10.1542/peds.2012-1672

[23] Katz T, Schatz J, Roberts CW. Comorbid obstructive sleep apnea and increased risk for sickle cell disease morbidity. Sleep Breath. 2018;22(3):797–804. 29450676 10.1007/s11325-018-1630-x

[24] Rosen CL, Debaun MR, Strunk RC, Redline S, Seicean S, Craven DI, et al. Obstructive sleep apnea and sickle cell anemia. Pediatrics. 2014;134(2):273–81. 25022740 10.1542/peds.2013-4223 PMC4187233

[25] Strauss T, Sin S, Marcus CL, Mason TBA, McDonough JM, Allen JL, et al. Upper airway lymphoid tissue size in children with sickle cell disease. Chest. 2012;142(1):94–100. 22241762 10.1378/chest.11-2013 PMC3419377

[26] Al-Otaibi T, Al-Qwaiee M, Faraidi H, Batniji F, Al-Otaibi F, Al-Harbi A. Prevalence of obstructive sleep apnea in children with sickle cell disease at a tertiary hospital in Saudi Arabia. Saudi Med J. 2017;38(6):616–20. 28578441 10.15537/smj.2017.6.19492 PMC5541185

[27] Raghunathan VM, Whitesell PL, Lim SH. Sleep-disordered breathing in patients with sickle cell disease. Ann Hematol. 2018;97(5):755–62. 29214337 10.1007/s00277-017-3199-z

[28] Atkeson A, Yeh SY, Malhotra A, Jelic S. Endothelial function in obstructive sleep apnea. Prog Cardiovasc Dis. 2009;51(5):351–62. 19249441 10.1016/j.pcad.2008.08.002 PMC4329644

[29] Ip MS, Tse HF, Lam B, Tsang KW, Lam WK. Endothelial function in obstructive sleep apnea and response to treatment. Am J Respir Crit Care Med. 2004;169(3):348–53. 14551167 10.1164/rccm.200306-767OC

[30] Nieto FJ, Herrington DM, Redline S, Benjamin EJ, Robbins JA. Sleep apnea and markers of vascular endothelial function in a large community sample of older adults. Am J Respir Crit Care Med. 2004;169(3):354–60. 14551166 10.1164/rccm.200306-756OC

[31] Chervin RD, Hedger K, Dillon JE, Pituch KJ. Pediatric sleep questionnaire (PSQ): validity and reliability of scales for sleep-disordered breathing, snoring, sleepiness, and behavioral problems. Sleep Med. 2000;1(1):21–32. 10733617 10.1016/s1389-9457(99)00009-x

[32] Chervin RD, Weatherly RA, Garetz SL, Ruzicka DL, Giordani BJ, Hodges EK, et al. Pediatric sleep questionnaire: prediction of sleep apnea and outcomes. Arch Otolaryngol Head Neck Surg. 2007;133(3):216–22. 17372077 10.1001/archotol.133.3.216

[33] Pandi-Perumal SR, Spence DW, BaHammam AS. Polysomnography: an overview. In: Pagel J, Pandi-Perumal S, editors. Primary care sleep medicine. New York: Springer; 2014.

[34] Berry RB, Budhiraja R, Gottlieb DJ, Gozal D, Iber C, Kapur VK, et al. Rules for scoring respiratory events in sleep: update of the 2007 AASM Manual for the Scoring of Sleep and Associated Events. Deliberations of the Sleep Apnea Definitions Task Force of the American Academy of Sleep Medicine. J Clin Sleep Med. 2012;8(5):597–619. 23066376 10.5664/jcsm.2172 PMC3459210

[35] Kapur VK, Auckley DH, Chowdhuri S, Kuhlmann DC, Mehra R, Ramar K, et al. Clinical Practice Guideline for Diagnostic Testing for Adult Obstructive Sleep Apnea: An American Academy of Sleep Medicine Clinical Practice Guideline. J Clin Sleep Med. 2017;13(3):479–504. 28162150 10.5664/jcsm.6506 PMC5337595

[36] Chan YH. Biostatistics 102: quantitative data–parametric & non-parametric tests. Singapore Med J. 2003;4(8):391–6. 14700417

[37] Chan YH. Biostatistics 103: qualitative data - tests of independence. Singapore Med J. 2003;44(10):498–503. 15024452

[38] Chan YH. Biostatistics 104: correlational analysis. Singapore Med J. 2003;44(12):614–9. 14770254

[39] Gillespie ML, Spring MR, Cohen RT, Klings ES. The interplay of sleep-disordered breathing, nocturnal hypoxemia, and endothelial dysfunction in sickle cell disease. Prog Pediatr Cardiol. 2023;68: 101602.

[40] Zahran AM, Nafady A, Saad K, Hetta HF, Abdallah AM, Abdel-Aziz SM, et al. Effect of Hydroxyurea Treatment on the Inflammatory Markers Among Children With Sickle Cell Disease. Clin Appl Thromb Hemost. 2020;26:1076029619895111. 31942811 10.1177/1076029619895111 PMC7098201

[41] Lanaro C, Franco-Penteado CF, Albuqueque DM, Saad ST, Conran N, Costa FF. Altered levels of cytokines and inflammatory mediators in plasma and leukocytes of sickle cell anemia patients and effects of hydroxyurea therapy. J Leukoc Biol. 2009;85(2):235–42. 19004988 10.1189/jlb.0708445

[42] Adegoke SA, Smith OS, Adekile AD, Figueiredo MS. Relationship between serum 25-hydroxyvitamin D and inflammatory cytokines in paediatric sickle cell disease. Cytokine. 2017;96:87–93. 28390266 10.1016/j.cyto.2017.03.010

[43] Silva-Junior AL, Garcia NP, Cardoso EC, Dias S, Tarragô AM, Fraiji NA, et al. Immunological Hallmarks of Inflammatory Status in Vaso-Occlusive Crisis of Sickle Cell Anemia Patients. Front Immunol. 2021;12: 559925. 33776989 10.3389/fimmu.2021.559925 PMC7990896

[44] da Silva RR, Pereira MC, Melo Rêgo MJ, Domingues Hatzlhofer BL, da Silva AA, Cavalcanti Bezerra MA, et al. Evaluation of Th17 related cytokines associated with clinical and laboratorial parameters in sickle cell anemia patients with leg ulcers. Cytokine. 2014;65(2):143–7. 24373941 10.1016/j.cyto.2013.11.012

[45] Ramos-Machado V, Ladeia AM, Dos Santos TR, da Anunciação FT, Terse-Ramos R. Sleep disorders and endothelial dysfunction in children with sickle cell anemia. Sleep Med. 2019;53:9–15. 30384138 10.1016/j.sleep.2018.08.019

[46] Kaleyias J, Mostofi N, Grant M, Coleman C, Luck L, Dampier C, et al. Severity of obstructive sleep apnea in children with sickle cell disease. J Pediatr Hematol Oncol. 2008;30(9):659–65. 18776757 10.1097/MPH.0b013e31817eb7ef

[47] Ferry AM, Wright AE, Ohlstein JF, Khoo K, Pine HS. Efficacy of a Pediatric Sleep Questionnaire for the Diagnosis of Obstructive Sleep Apnea in Children. Cureus. 2020;12(12): e12244. 33500863 10.7759/cureus.12244 PMC7819429

[48] Rosen CL, Wang R, Taylor HG, Marcus CL, Katz ES, Paruthi S, et al. Utility of symptoms to predict treatment outcomes in obstructive sleep apnea syndrome. Pediatrics. 2015;135(3):e662–71. 25667240 10.1542/peds.2014-3099 PMC4338327

[49] Capdevila OS, Kheirandish-Gozal L, Dayyat E, Gozal D. Pediatric obstructive sleep apnea: complications, management, and long-term outcomes. Proc Am Thorac Soc. 2008;5(2):274–82. 18250221 10.1513/pats.200708-138MG PMC2645258

[50] Salles C, Ramos RT, Daltro C, Barral A, Marinho JM, Matos MA. Prevalence of obstructive sleep apnea in children and adolescents with sickle cell anemia. J Bras Pneumol. 2009;35(11):1075–83. 20011842 10.1590/s1806-37132009001100004

[51] Eng J, Apergis GA, Fahmy S. Sickle cell disease is a risk factor for obstructive sleep apnea. Chest. 2010;138(4):322A-A.

[52] Rogers VE, Lewin DS, Winnie GB, Geiger-Brown J. Polysomnographic characteristics of a referred sample of children with sickle cell disease. J Clin Sleep Med. 2010;6(4):374–81. 20726287 PMC2919669

[53] Al Jabr I, Althabit F, Alonayzan A, Alsalman M. Risk of obstructive sleep apnea syndrome among adult patients with sickle cell disease in Saudi Arabia. IJMDC. 2021;5(1):93–7.

[54] Schellenberg JB, Maislin G, Schwab RJ. Physical findings and the risk for obstructive sleep apnea. The importance of oropharyngeal structures. Am J Respir Crit Care Med. 2000;162(2 Pt 1):740–8. 10934114 10.1164/ajrccm.162.2.9908123

[55] Schwab J. Sex differences and sleep apnoea. Thorax. 1999;54(4):284–5. 10092685 10.1136/thx.54.4.284 PMC1745455

[56] Bixler EO, Vgontzas AN, Lin HM, Liao D, Calhoun S, Vela-Bueno A, et al. Sleep disordered breathing in children in a general population sample: prevalence and risk factors. Sleep. 2009;32(6):731–6. 19544748 10.1093/sleep/32.6.731 PMC2690559

[57] Beebe DW, Rausch J, Byars KC, Lanphear B, Yolton K. Persistent snoring in preschool children: predictors and behavioral and developmental correlates. Pediatrics. 2012;130(3):382–9. 22891224 10.1542/peds.2012-0045 PMC3428758

[58] Lin CM, Davidson TM, Ancoli-Israel S. Gender differences in obstructive sleep apnea and treatment implications. Sleep Med Rev. 2008;12(6):481–96. 18951050 10.1016/j.smrv.2007.11.003 PMC2642982

[59] Feld L, Bhandari A, Allen J, Saxena S, Stefanovski D, Afolabi-Brown O. The impact of obstructive sleep apnea in children with sickle cell disease and asthma. Pediatr Pulmonol. 2023;58(11):3188–94. 37606223 10.1002/ppul.26643

[60] Wun T. The Role of Inflammation and Leukocytes in the Pathogenesis of Sickle Cell Disease. Hematology. 2000;5(5):403–12. 27420932 10.1080/10245332.2000.11746536

[61] Ahmed SG, Ibrahim UA, Hassan AW. Hematological parameters in sickle cell anemia patients with and without priapism. Ann Saudi Med. 2006;26(6):439–43. 17143019 10.5144/0256-4947.2006.439 PMC6074330

[62] Okpala I. The intriguing contribution of white blood cells to sickle cell disease: a red cell disorder. Blood Rev. 2004;18(1):65–73. 14684149 10.1016/s0268-960x(03)00037-7

[63] Wu M, Zhou L, Zhu D, Lai T, Chen Z, Shen H. Hematological indices as simple, inexpensive and practical severity markers of obstructive sleep apnea syndrome: a meta-analysis. J Thorac Dis. 2018;10(12):6509–21. 30746195 10.21037/jtd.2018.10.105 PMC6344721

[64] Barceló A, Morell-Garcia D, Sanchís P, Peña-Zarza JA, Bauça JM, Piérola J, et al. Prothrombotic state in children with obstructive sleep apnea. Sleep Med. 2019;53:101–5. 30504083 10.1016/j.sleep.2018.09.022

[65] De Franceschi L, Cappellini MD, Olivieri O. Thrombosis and sickle cell disease. Semin Thromb Hemost. 2011;37(3):226–36. 21455857 10.1055/s-0031-1273087

[66] Wang Y, Chen Y, Lin W, Huang M, Xu Y, Chen G. Inflammatory markers in children with obstructive sleep apnea syndrome. Front Pediatr. 2023;11: 1134678. 37114011 10.3389/fped.2023.1134678 PMC10127118

[67] Vgontzas AN, Bixler EO, Chrousos GP. Sleep apnea is a manifestation of the metabolic syndrome. Sleep Med Rev. 2005;9(3):211–24. 15893251 10.1016/j.smrv.2005.01.006

[68] Ryan S, Taylor CT, McNicholas WT. Predictors of elevated nuclear factor-kappaB-dependent genes in obstructive sleep apnea syndrome. Am J Respir Crit Care Med. 2006;174(7):824–30. 16840748 10.1164/rccm.200601-066OC

[69] Minoguchi K, Tazaki T, Yokoe T, Minoguchi H, Watanabe Y, Yamamoto M, et al. Elevated production of tumor necrosis factor-alpha by monocytes in patients with obstructive sleep apnea syndrome. Chest. 2004;126(5):1473–9. 15539715 10.1378/chest.126.5.1473

[70] Ming H, Tian A, Liu B, Hu Y, Liu C, Chen R, et al. Inflammatory cytokines tumor necrosis factor-alpha, interleukin-8 and sleep monitoring in patients with obstructive sleep apnea syndrome. Exp Ther Med. 2019;17(3):1766–70. 30783447 10.3892/etm.2018.7110 PMC6364239

[71] Vilas-Boas W, Veloso Cerqueira BA, Figueiredo CV, Santiago RP, da Guarda CC, Pitanga TN, et al. Association of homocysteine and inflammatory-related molecules in sickle cell anemia. Hematology. 2016;21(2):126–31. 26334689 10.1179/1607845415Y.0000000048

[72] Anderson ME Jr, Brancazio B, Mehta DK, Georg M, Choi SS, Jabbour N. Preferred parental method of post-operative tonsillectomy and adenoidectomy follow-up (phone call vs. clinic visit). Int J Pediatr Otorhinolaryngol. 2017;92:181–5. 28012526 10.1016/j.ijporl.2016.11.025

[73] Setty BN, Stuart MJ, Dampier C, Brodecki D, Allen JL. Hypoxaemia in sickle cell disease: biomarker modulation and relevance to pathophysiology. Lancet. 2003;362(9394):1450–5. 14602439 10.1016/S0140-6736(03)14689-2

[74] Valrie CR, Bromberg MH, Palermo T, Schanberg LE. A systematic review of sleep in pediatric pain populations. J Dev Behav Pediatr. 2013;34(2):120–8. 23369958 10.1097/DBP.0b013e31827d5848 PMC3562475

