# Supplementary figures and images for: Role of endothelial dysfunction in sleep-disordered breathing in egyptian children with sickle cell disease
Source: BMC Pediatr. 2024 Oct 1;24:626. doi: 10.1186/s12887-024-05066-6 (PMC11443814; doi:10.1186/s12887-024-05066-6)

**Pediatric Sleep Questionnaire**

**
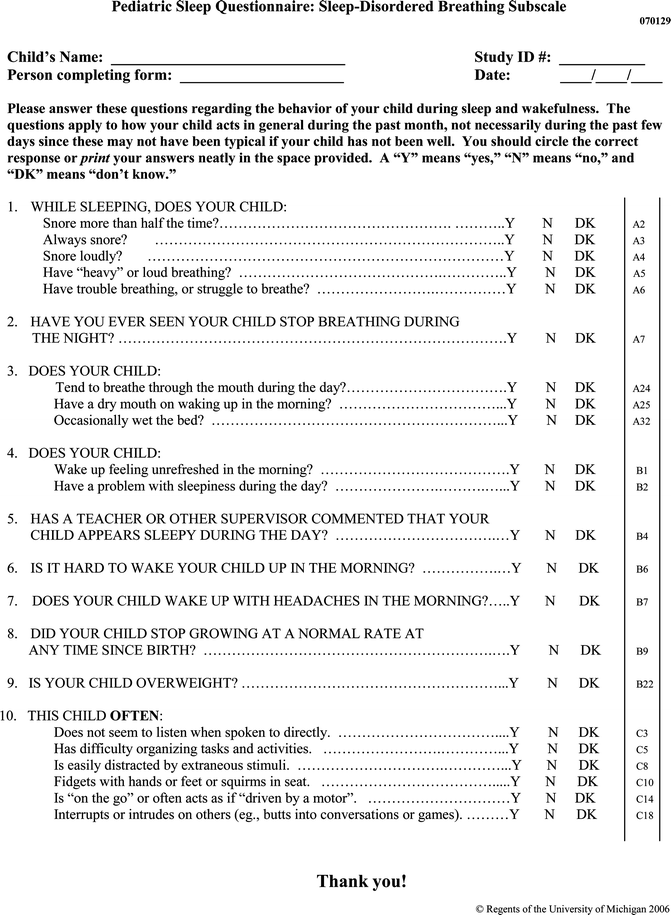
**

Supplement: Supplementary file 1 — Supplementary Material 1 [file 12887_2024_5066_MOESM1_ESM.docx]
